# Gliadin-Specific T-Cells Mobilized in the Peripheral Blood of Coeliac Patients by Short Oral Gluten Challenge: Clinical Applications

**DOI:** 10.3390/nu7125515

**Published:** 2015-12-02

**Authors:** Stefania Picascia, Roberta Mandile, Renata Auricchio, Riccardo Troncone, Carmen Gianfrani

**Affiliations:** 1 Institute of Protein Biochemistry-CNR, Via Pietro Castellino 111, Naples 80131, Italy; s.picascia@ibp.cnr.it; 2 Department of Translational Medical Science (DISMET), Section of Pediatrics, University of Naples Federico II, Via S Pansini 5, Naples 80131, Italy; ro.mandile@libero.it (R.M.); r.auricchio@unina.it (R.A.); troncone@unina.it (R.T.); 3 European Laboratory for the Investigation of Food-Induced Diseases (ELFID), University of Naples Federico II, Via S Pansini 5, Naples 80131, Italy

**Keywords:** celiac disease, gluten challenge, interferon-γ, ELISPOT

## Abstract

Celiac disease (CD) is a common lifelong food intolerance triggered by dietary gluten affecting 1% of the general population. Gliadin-specific T-cell lines and T-cell clones obtained from intestinal biopsies have provided great support in the investigation of immuno-pathogenesis of CD. In the early 2000 a new *in vivo*, less invasive, approach was established aimed to evaluate the adaptive gliadin-specific T-cell response in peripheral blood of celiac patients on a gluten free diet. In fact, it has been demonstrated that three days of ingestion of wheat-containing food induces the mobilization of memory T lymphocytes reactive against gliadin from gut-associated lymphoid tissue into peripheral blood of CD patients. Such antigen-specific T-cells releasing interferon-γ can be transiently detected by using the enzyme-linked immunospot (ELISPOT) assays or by flow cytometry tetramer technology. This paper discusses the suitability of this *in vivo* tool to investigate the repertoire of gluten pathogenic peptides, to support CD diagnosis, and to assess the efficacy of novel therapeutic strategies. A systematic review of all potential applications of short oral gluten challenge is provided.

## 1. Introduction

Celiac Disease (CD) is one of the most common food intolerances affecting almost 1% of worldwide population [1]. The disease develops in genetically predisposed subjects as a consequence of an abnormal immune response to wheat gluten and related prolamines of rye and barley. A decisive role in the pathogenesis is played by intestinal gliadin-specific T-cells whose presence seems to be specific of CD patients. Though the causative factor is a dietary protein, CD is considered a chronic inflammatory disorder characterized by autoimmune features. In fact, virtually all subjects with CD produce antibodies against the tissue transglutaminase (tTG) of IgA type which are the disease hallmark with diagnostic relevance [2].

For decades, CD has been considered prevalently an intestinal disease, and the enteropathy the main clinical and histological outcome. Accordingly, the evaluation of small intestinal histology has been for many years the only diagnostic tool in CD [3]. However, the high specificity and sensitivity of tTG IgA antibodies has recently led to a revision of the diagnostic criteria, especially for pediatric subjects. Based on these new guidelines from the ESPGHAN (The European Society for Paediatric Gastroenterology Hepatology and Nutrition), the evaluation of intestinal mucosa should be no more necessary to make a diagnosis of CD in the presence of clear symptoms, genetics, and high anti-tTG titers [4]. Although subjects with overt CD also have a high level of antibodies against gliadin, either for native (AGA) and deamidated (DGP) gliadin peptides, the AGA are not recommended in the diagnosis of CD due to their low sensitivity and specificity. By contrast, the DGP-IgG have a higher specificity and are recommended for the CD diagnosis in case of IgA deficiency, or in patients with both anti-tTG and anti-endomysium (EMA) negative serology. Furthermore, the use of DGP is suggested especially for patients younger than two years. Notwithstanding, a diagnostic challenge is still posed for those patients deliberately on gluten-free diets to which the intestinal histology and serum antibodies are not helpful. For these specific cases, and for other situations of diagnostic uncertainty, there is still a demand of novel approaches to make a clear and undoubted diagnosis of CD.

For both diagnostic purposes, and to study the mechanisms leading to CD, the demonstration and characterization of gliadin-specific, pathogenic T-cell response is mandatory. In the early 2000s, Anderson and co-workers established an *in vivo* approach to detect in peripheral blood the gluten-specific T-cells of intestinal origin by using the sensitive enzyme-linked immunospot (ELISPOT) assay, widely and successfully used to study antigen-specific T cells secreting cytokines, as well as antibody-producing B cells, particularly in infectious diseases [5]. The procedure developed by Anderson and co-workers requires the oral administration of wheat bread for three days to celiac patients on strict gluten free diet (GFD) and the collection of blood samples soon before and six days after the challenge started [6]. Since its first application, several studies have shown that the short gluten challenge (SGC) quickly mobilizes T-cells in the blood of celiac patients on GFD that can be revealed by interferon-γ ELISPOT assays, or flow cytometry tetramers technology, thus suggesting its great clinical potentiality. In general, the clinical symptoms are not severe, and the serum levels of CD-associated antibodies are unchanged, though in some patients morphological changes can occur after three days of the gluten challenge [7,8,9,10].

## 2. Genetic Susceptibility, Clinical Spectrum, and Pathogenesis

The susceptibility to develop celiac disease is strongly influenced by inherited factors. The Human Leukocyte Antigen (HLA) class II genes encoding for DQ2.5 (DQA1*05 and DQB1*02 alleles) and for DQ8 heterodimers (DQA1*03 and DQB1*0301 alleles) are the main risk factors [11]. Although more than 90% of patients with celiac disease have the DQ2.5 genotype, and the remaining ones carry either the DQ2.2 or the DQ8 genes, HLA class II account for about 40% of the genetic risk in CD [12,13,14]. Genome wide association studies (GWAS) have recently identified two genes, the B08 and B39 of the HLA class I locus, and a large number of non-HLA genes associated to CD, almost all of them involved in the inflammatory pathways [15].

In CD patients the dietary ingestion of wheat gluten activates a strong immune response characterized by the lymphocytic infiltration in the proximal part of the small bowel [11]. Gluten-activated T lymphocytes populate both the epithelium and lamina propria, and play a key role in damaging the intestinal mucosa [11,16]. The consequence is the villous atrophy and crypt hyperplasia that occurs within a variable window of time after the first gluten consumption. The intestinal damage can range from very mild, showing little or absent histological intestinal lesions, to a complete villous flattening, according to Marsh-Oberhuber classification [17]. From the clinical point of view, CD can present in different forms [18]. In the “classical” form, the ingestion of gluten induces an enteropathy mainly characterized by signs of malabsorption with different degrees of villous atrophy. Most common in adult age, CD may have a “non-classical” form, with no weight loss, nor classical symptoms. The disease may even be “subclinical”, with no symptoms albeit in the presence of a villous atrophy. In addition, there are genetically predisposed individuals who have high anti-tTG titers, but normal small bowel mucosa. It has been reported that almost one third of these individuals with “potential celiac disease” will develop the overt disease within nine years [19].

Recent evidence has highlighted that the number of gluten-reactive T cells both in peripheral blood and in the small intestinal biopsy of CD patients positively correlated with the degree of histological intestinal damage. Similarly, the serum anti-TG IgA antibody levels have been found to significantly correlate to the Marsh grade of mucosal damage [16,20].

Furthermore, it is well known that T lymphocytes reacting to specific gluten peptides and releasing inflammatory cytokines, such as IFN-γ and IL-21, reside in the intestinal mucosa of subjects with CD but not in healthy controls [21]. These cells, mainly CD4+T lymphocytes, react to long fragments (up to 30–40 amino acid residues) of gluten resistant to gastrointestinal enzymatic degradation. These gluten peptides pass through the epithelial barrier via transcellular [22] or paracellular transport [23,24], this latter favored by an increased epithelial permeability mediated by the release of zonulin, an intestinal peptide that is involved in the tight junction regulation [25]. When in the *lamina propria* compartment, the gluten peptides become substrate for the enzyme tissue transglutaminase type 2 (tTG2) [26]. In particular stress conditions, the tTG2 is released in the extracellular matrix, and acquires an open active form [27]. After the activation, tTG2 specifically converts glutamine residues (neutrally charged) in glutamic acid (negatively charged) residues. The deamidated peptides fit the binding pockets of both DQ2 and DQ8 molecules, having a strong affinity for negative charged peptides [28]. As a consequence, the complex gluten peptide-HLA DQ2/DQ8 is specifically recognized by CD4+ T lymphocytes bearing the α/β T-cell receptor (TCR) and activating the inflammatory cascade.

The great heterogeneity of gluten proteins accounts for the large diversity of T-cell epitopes found to be active in celiac patients [29,30]. The identification of a complete repertoire of gluten immunogenic sequences is mandatory to better understand either CD pathogenesis, and to provide the bases for specific disease-targeted immuno-modulatory treatments. Among the several immunogenic sequences, three peptides were found the most active: the 33-mer from the α-gliadin (containing the DQ2.5-glia-α1a, DQ2.5-glia-α2 epitopes); the 17-mer from ω-gliadin (containing the DQ2.5-glia-ω-1, DQ2.5-glia-ω-2 epitopes); and the γ-gliadin DQ2.5-glia-γ-1 epitope [31,32,33,34]. Of note, many of the gluten T-cell stimulatory sequences have been identified thanks to the availability of stable T-cell lines and T-cell clones raised from intestinal mucosa tissues. However, the intestinal T-cell cultures have several technical restrictions mainly due to: (i) the limited numbers of cells that can be obtained from intestinal biopsies, (ii) long time necessary to establish growing T-cell cultures. Because of that, there is the need to find new tools that allow to investigate the gluten-specific CD4+ T-cell response in CD.

## 3. Current and Emerging Therapies

To date, the only valid treatment for celiac patients is the GFD [35], based on the strict avoidance of wheat, rye, barley, and all related cereals, including spelt (a wheat variant). After a strict GFD, the intestine recovers a normal morphology and function, and concomitantly, all symptoms and serological disease markers disappear. If from one side the GFD allows the restoration of the intestinal physiological function, from the other side it is expensive and provides several social restrictions, and compliance to GFD is not optimal, particularly in adolescence [36,37]. Nutritional properties of gluten free foods, as for example the high glycemic index and caloric power, increase the risk of treated celiacs to develop nutritional alterations, obesity, or metabolic syndromes [38,39]. In addition, there is a minority of patients that suffers from a refractory condition, in which the diet is not efficacious, and requires a pharmacological, anti-inflammatory treatment [40,41]. A deeper knowledge of CD pathophysiology has opened to the investigation of several therapeutic drug-based approaches in the last decade, some of them currently on clinical trial phase II to assess their efficacy [42]. This promising scenario strongly demands the availability of a rapid, safe, and reproducible *in vivo* assay to assess the efficacy of emerging novel therapies to treat CD [43].

## 4. Gluten Oral Challenge as Tool to Monitor Intestinal Gluten-Reactive T-Cells

The gluten challenge is a clinical approach widely used in the last decades to have a diagnosis of celiac disease. It consists in the introduction of gluten containing foods in subjects previously on a gluten free diet, for a time frame necessary to provoke a clinical response (from two weeks up to four months). About 75% of adults received a clear diagnosis in at least two weeks [44], however the response rates and the onset of symptoms were highly variable among different patients [45,46]. Either histological, serological, and symptomatic changes are evaluated, to monitor the efficacy of gluten challenge. However, the extensive gluten challenge has some limitations, such as the risk of the overt disease induction (especially in younger patients), and the invasive endoscopy as final exam. Altogether, these findings have raised the need of alternative, less invasive, procedures to investigate the role of gluten-specific T-cells in the pathogenesis of CD.

### 4.1. Interferon-γ ELISPOT Assay on Peripheral Blood Cells after a Three Day Gluten Challenge

For long time, all the efforts to isolate gliadin-specific memory T-cells from peripheral blood samples of CD patients gave poor results, due to the low frequency in the blood of gluten-primed intestinal CD4+ T-cells, and to a substantial functional differences (in particular a diverse HLA restriction) that has been reported between gliadin-specific T-cell clones raised from the gut or blood [26,47,48]. As consequence, peripheral blood samples have, for a long time, been considered not optimal tissue material to study the anti-gluten T-cell immunity. At the beginning of this century, Anderson and co-workers published a study describing a new *in vivo* approach to analyze T-cell response to gluten in peripheral blood, overcoming in this way all the technical problems related to the use of intestinal T-cells [6]. This procedure requires the oral administration of bread slices (approximately 200 g/day) for three days to CD patients on a strict GFD, and blood samples obtained at different time points during the gluten challenge. The gluten-reactive T-cells are monitored in peripheral blood mononuclear cells (PBMCs) by detecting those releasing IFN-γ, the prominent mediator of the inflammatory cascade in celiac mucosa, by ELISPOT assay. The ELISPOT is a sensitive technique able to catch single cell secreting cytokine, or other immune mediators, upon specific stimuli. In this pilot study, Anderson and co-workers found that the SGC rarely causes problems, as only few volunteers, out of 16 adult CD patients enrolled, showed clinical symptoms of disease (usually mild), or had histological signs of intestinal mucosa inflammation. The classical serological markers of CD, as the anti-endomysium and anti-tTG2 antibodies, remained negative after the SGC. The gluten challenge induced in celiac patients a transient IFN-γ response to tTG-treated chymotrypsin-digested gliadin that was maximum six days after the volunteers began eating bread slices. A 660% increment of IFN-γ spot-forming cells (IFN-γ-SFC) was reported at day six compared to responses before the challenge, whilst only a 37% of IFN-γ-SFC increment was detected in DQ2+ control group. In addition, the gut origin of these circulating T lymphocytes, mobilized in response to the gluten challenge, was supported by the expression of the α4β7 integrin [7,8], a classical marker of gut homing [49,50]. Similarly, the restriction of gluten response by HLA class II DQ2 molecules, associated to CD risk, was also demonstrated.

A subsequent study from our group performed in adolescent CD patients has reported that the SGC is a reproducible assay. To further demonstrate that the SGC is a valid instrument to investigate the gluten induced immune response, 14 young celiac patients on GFD underwent two separate gluten consumptions, with the same procedure described by Anderson *et al.* After three to five months of gluten wash-out, the celiac cohort underwent a second cycle of wheat-containing food challenge. We found that the IFN-γ responses significantly increased in peripheral blood sampled six days after the second challenge, and interestingly, gliadin reactive cells were more frequent compared to the first challenge, most likely due to the increased frequency of memory T-cells activated upon the first gluten exposure [8].

### 4.2. Interferon-γ ELISA Assay and Multiparametric Masscytometry to Monitor the Anti-Gluten T-Cells Response after a Three Day Gluten Challenge

Other studies have reported *in vitro* read-outs different from ELISPOT assay to assess the specific immune response elicited by gluten challenge. Ontiveros *et al.* have developed a whole blood assay to detect gluten-specific T-cells by dosing IFN-γ in the serum by ELISA after stimulation of blood with gluten/peptides [51]. The same research group has also analyzed the peripheral blood cell response to gluten upon the three days of wheat consumption by measuring the cell proliferation and found results consistent with the IFN-γ ELISPOT findings [6].

Despite the central role given by HLA class II in CD, being the main genetic risk factor and the key restriction molecules of pathogenic CD4+ T-cells, studies from our group have demonstrated that gliadin contain peptides able to stimulate cytotoxic CD8+ T-cells in an antigen restricted manner when presented on surface of antigen presenting cells (APC), such as B- or enterocytes by HLA class I molecules [52]. Of note, a more recent study from Mark Davis and co-workers using the potent multiparametric CyTOF technology approach, that allows to monitor simultaneously more than 50 different T-cell markers, showed that the three days gluten challenge induced in peripheral blood of CD patients a remarkable increased of either TCRαβ- and TCRγδ- bearing CD8+ T lymphocytes, other than the CD4+ T-cells [53]. These lymphocytes, expressed the gut homing markers, such as CD103 (intestinal epithelial-homing markers αE) and β7-integrins, thus demonstrating their origin from intestinal mucosa. The percentage of each cell subset mobilized by gluten intake varies among single patients, but ranged from 1% up to 10% of total peripheral CD8+ cells. This keynote study has demonstrated that memory CD8+ T-cells are activated by the oral gluten challenge and circulate from the target intestinal tissue to peripheral blood. However, further studies are necessary to assess the gluten specificity of these CD8+ T cells mobilized by the SGC.

### 4.3. HLA-DQ2-Tetramers as Probe to Detect Gliadin-Specific Cells in Peripheral Blood

In the recent years, much attention has been paid to the use of tetramers technology to dissect specific T-cell responses to a variety of antigenic sources [20]. Tetramers are composed by four major histocompatibility complex (MHC) molecules each of them loaded with a single antigenic peptide, labelled with fluoresceinated biotin-streptavidin complex. The MHC-peptide construct binds to a single T-cell receptor on the surface membrane of cognate T-cells. When the tetramer is bound, the cells can be visualized by flow cytometry analysis [54]. This sensitive assay allows to quantify the cell frequency, to assess their phenotype, or to separate the cell subset that specifically reacts to a single antigen. Tetramer complexes have been widely and successfully used to study MHC class I-restricted CD8+ T lymphocytes specific for infectious diseases or tumor antigens [55].

DQ2-gliadin-tetramer tests were first used by Raki and co-workers to monitor CD4+ T lymphocyte specific for two immunodominant gluten epitopes, DQ2.5-glia-α1a and DQ2.5-glia-α2, in PBMCs of celiac patients underwent the SGC [10]. The response rate of such test (approximately 85% sensitive and 100% specific evaluated in HLA-DQ2.5+ celiac patients *vs.* HLA-matched controls) is comparable to that found in IFN-γ ELISPOT assay [10]. Frequencies of positive cells identified after gluten challenge is similar between the two approaches (number of IFN-γ secreting cells found by ELISPOT ranging from 1 to 5000 in comparison to DQ2.5-glia-α1a tetramer positive cells ranging 1:1000 and DQ2.5-glia-α2 tetramer positive cells 1:5000). Similarly to the IFN-γ ELISPOT findings, no tetramer positive cells were detected in DQ2+ healthy controls, either before or after the brief gluten exposure. Interestingly, in subjects with a diagnosis of CD, 5%–8% of total CD4+ cells were stained with tetramer specific for both DQ2.5 α epitopes [10]. More recently, other studies from the same group have monitored gluten-specific T-cells in peripheral blood of celiac patients by tetramer technology without the gluten oral challenge [20]. More specifically, gliadin-tetramer positive cells have been detected in peripheral blood of both treated and untreated DQ2-positive subjects with CD.

In addition, a single cell-TCR sequence analysis performed on DQ2-gliadin-tetramer specific T-cells, mobilized upon the gluten challenge, has demonstrated how highly focused the TCR repertoire is of CD4+ T-cells specific for the immunodominant gluten epitopes [53,56,57,58]. Collectively, all these studies demonstrated the great potentiality of the tetramer technology as a tool to investigate the anti-gluten T-cell responses. However, tetramer assay has both pros and cons. The main advantage is that it allows to quantify the antigen-specific cells independently by their immune function or activation state. More specifically, this technology can also monitor cells not releasing a specific cytokine [9]. However, despite the high sensitivity, tetramers allow the identification of only cells specific for a single peptide, whereas ELISPOT assay allows simultaneous monitoring for T cells reacting to a wider repertoire of gluten epitopes. Tetramer production, furthermore, is challenging, being laborious, expensive, and time consuming all factors that render this technology difficult in application, especially in a clinical practice context. Notwithstanding the above advantages or disadvantages, it is evident that larger cohort of patients and healthy controls are needed to validate the sensitivity of tetramer technology to diagnose CD, independently of the gluten challenge.

## 5. Translational Applications of the Short Gluten Oral Challenge

### 5.1. Identification of Gluten Immunogenic Peptides

Since the first description, the short oral gluten challenge has become an attractive tool for all researchers interested in the identification of the complete repertoire of gluten (and of other prolamin) toxic sequences [32,59] (Table 1). Tye-Din and co-workers found a high degree of T-cell peptide cross reactivity in adult celiacs underwent the SGC by screening a large library (almost 3000) of 20-mer peptides derived from gluten, hordein, and secalin [32]. Interestingly, though many peptides were immunogenic, only the T-cell clones specific for three peptides containing five epitopes (DQ2.5-glia-α1a/DQ2.5-glia-α2; DQ2.5-glia-ω-1/DQ2.5-glia-ω-2; DQ2.5-Hor-1) were found responsible for the great majority of responses in adult CD, thus demonstrating a high T-cell stimulatory peptide redundancy.

A recent study from Hardy and co-workers [60] has expanded such peptide repertoire analysis to a pediatric cohort of CD patients. A comparable pattern of peptide recognition was found between children and adult with CD. These similarities in the nature of the T-cells induced by the *in vivo* SGC between pediatric and adult CD can have a great potentiality for the applications also in celiac children of the peptide-based therapy designed for adults.

### 5.2. Validation of Therapeutic Drugs

Many studies aimed to identify new strategies to detoxify wheat gluten, and several of these are based on enzymatic technologies that degrade fragments or mask gluten immune-stimulatory sequences [34] (Table 1). The high content in proline and glutamine-rich peptides make gluten resistant to proteolysis by gastric, pancreatic, and intestinal brush border membrane enzymes. Partially digested gluten fragments stimulate the immune system and became toxic for celiac disease patients [11]. The identification of a combination of enzymes that can break proline and glutamine bounds is a fascinating goal for celiac researchers, and it represents an interesting future perspective for pharmaceutical sector that aims to produce oral drugs. To this specific purpose, several gluten-specific proteases, called glutenases, have been isolated from bacteria, fungi, and cereals and are currently under clinical trial investigation. ALV003 is a promising mixture of two glutenases which cleaves gluten fragments at site enriched in proline and glutamine: a cysteine-endoprotease derived from germinated barley seeds (EP-B2), able to breaks gluten protein, and a prolyl endopeptidase (PEP) from S. capsulate (SC-PEP) that cleaves proline residues. When combined in 1:1 ratio these two glutenases maximized the enzymatic activity [61]. In a clinical trial, 20 patients with celiac disease on GFD were randomized to eat either gluten (16 g/day for three days) pre-treated with ALV003, or gluten pre-treated with placebo. Patients who received ALV003 gluten had significantly lower peripheral T-cell IFN-γ response to the immunodominant α-gliadin 33-mer multi-epitope peptide, or whole gliadin, compared to the group that received the placebo [62]. The relevance of the SGC to monitor drug efficacy has been demonstrated in a follow-up study, where a double blind, placebo-controlled trial was performed on 41 adults CD patients randomized to assume ALV003, or the placebo, along with a gluten daily intake (2 g/day) for six weeks [63]. In this second study, the main clinical read-out was the evaluation of the small intestinal mucosa damage that appeared. Signs of lymphocytes activation, and intraepithelial infiltration of CD3+ lymphocytes, both TCRα/β and TCRγ/δ, were found significantly increased only in the placebo-treated patients, while these markers remained almost unchanged in ALV003 treated group. Though very promising, this drug shows an interesting expectative for its future application, further investigations are necessary to monitor the long term effects.

Other strategies have been developed to directly detoxify wheat flour, as the extensive hydrolysis during the sourdough fermentation with a mixture of acid bacteria proteases [64], or the transamidation with methyl-lysine of specific glutamines that are target of the tTG [65] (Table 1). The demonstration of the immuno-stimulatory properties of fermented or transamidated wheat after a short challenge may provide rapid and preliminary information about the safety and efficacy of these novel and promising strategies to produce wheat-based gluten free food for celiac disease.

### 5.3. Diagnostic Relevance

In recent times, a great attention has been paid to develop clinical practices less invasive than endoscopy to diagnose celiac disease (Table 1). Moreover, the increased attention paid by the general public to food related problems, as well as the improved distribution of gluten free foods, has spread the belief that gluten free diet coincides with a healthy life style; as a consequence more and more people voluntary exclude gluten from their diet without a clear diagnosis of celiac disease [66]. This makes difficult for clinicians to formulate a definitive diagnosis of celiac disease in unclear cases, as both serological and histological tests revert to the normal value on GFD. Even the HLA genotyping of such individuals complaining gluten related disorders, and on arbitrary GFD, does not help to make a definitive diagnosis of CD in DQ2 or DQ8 positive subjects, having this test only a negative prediction value. To date, the only instrument to practice a correct diagnosis in such doubtful cases consists in the evaluation of histological lesions after a long-term gluten challenge. A recent study conducted in adults has shown that at least two weeks of gluten consumption allow a clear diagnosis of celiac disease in over 75% of CD population [44]. However, the time frame of the gluten challenge is variable, as indicated by several studies, and may be longer than two weeks. In fact it is clear that sensitivity to gluten exposure varies greatly between coeliac patients and some may take much longer before showing signs of relapse [4,46]. This long-term gluten challenge makes this diagnostic procedure difficult to practice. Moreover a long-term challenge is not suggested in children younger than five years old and during a pubertal growth spurt. The short-time (only three days) of the gluten challenge and the sensitivity of immunological tests offer an interesting perspective for using the SGC as a diagnostic tool. Pilot studies of gluten challenge as diagnostic tool for subjects coming to the observation when they are already on gluten free diet came from Brottveit *et al.* [10]. The authors enrolled 35 subjects with uncertain diagnosis and on GFD for at least four weeks and 13 patients with treated CD. All the enrolled subjects underwent the SGC and endoscopy for biopsy sampling at day 0 and day 4 of the gluten challenge. By the positive detection of tetramers the authors have observed gliadin-specific T-cells in 11/13 CD patients, and in only 2/35 with uncertain CD, thus identifying a group of subjects clinically gluten-sensitive and yet negative to this test.

**Figure 1:**
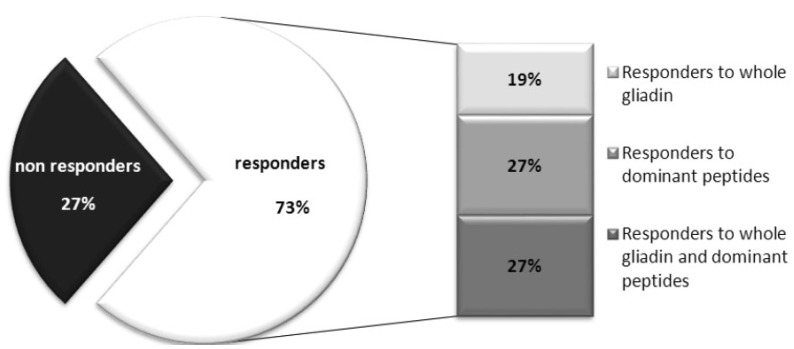
Percentage of subject responders to the short gluten challenge. A cohort of 36 Italian DQ2-positive young celiac patients [8,60] consumed 3–4 wheat bread slices (corresponding to 9–12 g of gluten/die) for three days. Immunoreactivity was evaluated in peripheral blood at day 0 and day 6 by INF-γ-ELISPOT assay, in response to either whole gliadin or immunodominant α-gliadin peptides.

In order to validate the SGC as a procedure to support diagnosis of celiac disease in uncertain cases, it is mandatory the assessment of its sensitivity and specificity. Regarding the sensitivity, there is a large variability in the cut-off applied to define subject responders to the gluten challenge among the different studies. In our recent experience of brief gluten challenge performed on a cohort of 36 DQ2+ CD adolescent celiacs [8,60], each single patient was considered responsive to oral challenge when showed levels of INF-γ secreting cells in response to whole gliadin, and/or dominant gliadin peptides, that exceeded two-fold the INF-γ responses at day 0 (Fold Increase-FI ≥ 2) and a difference of SFC/well (ΔSFC) of at least 10 between day 6 and day 0. Based on these criteria, we found that almost 73% of our 36 patients are responsive (Figure 1). Other studies reporting a higher frequency of DQ2+ CD responders (by ELISPOT) ranging from 85% to 92% of responder cases [6,7,8,51]. These un-matched results, obtained in different celiac cohorts, highlighted how it is important to identify common and unique criteria to validate unequivocally the subjects that positively respond to gluten challenge (responders), and to distinguish from the non-responder ones. It is evident that further work is necessary to validate the SGC as diagnostic tool for celiac disease; it is particularly fundamental to expand such an analysis to HLA DQ2+ non celiac healthy control to assess its specificity, which is to date poorly investigated. Indeed the classical diagnostic approach characterized by the long gluten challenge, and monitored by serology and histology, is more stable and reliable than the brief challenge, despite that there is the need to have a shorter and less invasive test to apply to the clinical practice. As stated above, the tetramers are promising diagnostic tools, however several technological limits still remain to be solved for their use large-scale.

**Table 1:** Possible translational applications of brief gluten challenge.

Application	Clinical/Research Purpose	References
Diagnosis	Confirmation of diagnosis in uncertain celiac disease cases	[10,67]
Therapies	Validation of new therapeutic drugs	[62]
Therapies	Validation of biochemical or enzymatic strategies to detoxify gluten.	[64,65]
Therapies	Searching of wheat cultivars with reduced immunotoxic gluten sequences	[68]
Pathogenesis	Identification of immunogenic gluten epitopes	[32,60]
Pathogenesis	Phenotypic analysis of cell population involved in celiac disease	[53]

## 6. Conclusions

The short (three days) gluten challenge is a validated tool for the evaluation and monitoring in peripheral blood of gluten-specific T-cell response that are elicited in the gut after gluten exposure. Since its first demonstration in early 2000 [6], this *in vivo* procedure, less invasive than the endoscopy, has allowed the screening of large peptide library and provided a great help for the characterization of a repertoire of immunostimulatory gluten sequences, a fundamental step to constructing a peptide-base immunotherapy for the treatment of CD [29,32]. Furthermore, the short oral challenge has been a powered instrument to demonstrate that gluten mobilizes intestinal CD8+ T-cells, corroborating their role in CD pathogenesis. The SGC is a promising tool to assess the efficacy of novel treatments aimed to reduce the load of toxic gluten or of immunomodulatory drugs.

Finally, thanks to several sensitive assays to measure anti-gluten T response in blood cells, as IFN-γ ELISPOT/ELISA or tetramers-flow cytometry technologies, it is thinkable to apply this innovative approach to clinical practice, in order to help specialists in making a correct and definitive diagnosis of celiac disease in those cases in which subjects are arbitrarily on gluten free diet, or can help the diagnosis in case of potential celiac disease. To make this technique adapt to clinical practice, several parameters still have to be addressed. Further work is necessary to reach a high sensitivity, to keep lowest number of false negative subjects. So far, the main limitations playing against the wide use of oral challenge for clinical practice are less sensitivity and specificity compared to available serology tests, and the high cost of ELISPOT and tetramers immune assays. Notwithstanding, these limitations do not lessen the value of the oral challenge, as a rapid tool to assess the efficacy of several alternative therapies currently under investigation.
